# Introduction history overrides social factors in explaining genetic structure of females in Mediterranean mouflon

**DOI:** 10.1002/ece3.3433

**Published:** 2017-11-16

**Authors:** Elodie Portanier, Mathieu Garel, Sébastien Devillard, Pascal Marchand, Julie Andru, Daniel Maillard, Gilles Bourgoin

**Affiliations:** ^1^ Laboratoire de Biométrie et Biologie Evolutive CNRS Université Claude Bernard Lyon 1 Université de Lyon Villeurbanne France; ^2^ Unité Faune de Montagne Office National de la Chasse et de la Faune Sauvage Juvignac France; ^3^ VetAgro Sup ‐ Campus Vétérinaire de Lyon Université de Lyon Marcy l'Etoile France

**Keywords:** introduction, large herbivores, *Ovis*, socio‐spatial organization, spatial genetic structure

## Abstract

Fine‐scale spatial genetic structure of populations results from social and spatial behaviors of individuals such as sex‐biased dispersal and philopatry. However, the demographic history of a given population can override such socio‐spatial factors in shaping genetic variability when bottlenecks or founder events occurred in the population. Here, we investigated whether socio‐spatial organization determines the fine‐scale genetic structure for both sexes in a Mediterranean mouflon (*Ovis gmelini musimon *× *Ovis* sp.) population in southern France 60 years after its introduction. Based on multilocus genotypes at 16 loci of microsatellite DNA (*n* = 230 individuals), we identified three genetic groups for females and two for males, and concurrently defined the same number of socio‐spatial units using both GPS‐collared individuals (*n* = 121) and visual resightings of marked individuals (*n* = 378). The socio‐spatial and genetic structures did not match, indicating that the former was not the main driver of the latter for both sexes. Beyond this structural mismatch, we found significant, yet low, genetic differentiation among female socio‐spatial groups, and no genetic differentiation in males, with this suggesting female philopatry and male‐biased gene flow, respectively. Despite spatial disconnection, females from the north of the study area were genetically closer to females from the south, as indicated by the spatial analysis of the genetic variability, and this pattern was in accordance with the common genetic origin of their founders. To conclude, more than 14 generations later, genetic signatures of first introduction are not only still detectable among females, but they also represent the main factor shaping their present‐time genetic structure.

## 1 INTRODUCTION

In the wild, individuals are not randomly distributed across the landscape, leading to various levels of spatial structure among and within populations (Hedrick, 2011; Sugg et al., 1996). Spatial structure may result from environmental constraints (e.g., patchy environment or presence of barriers, Epps et al., 2005), social organization (e.g., family groups or philopatry, Hazlitt, Eldridge, & Goldizen, 2004; Perrin, Allaine, & Le Berre, 1993; Storz, 1999), and demographic history (e.g., introductions, Biebach & Keller, 2009; Simpson et al., 2013). Depending on the behavioral characteristics of a species (e.g., philopatry, dispersal, migration), such a spatial structure can play an important role in determining gene flow, and consequently the genetic structure of a population (Slatkin, 1987). Among other benefits, gene flow often contribute to maintain genetic diversity and heterozygosity in populations (Garant, Forde, & Hendry, 2007; Říčanová et al., 2011; Segelbacher et al., 2010), which in turn limit inbreeding depression (Keller & Waller, 2002) and preserve both immunocompetence (Kloch et al., 2013; MacDougall‐Shackleton et al., 2005) and adaptability of populations to changing environments (Frankham, Ballou, & Briscoe, 2004). Investigating gene flow is therefore of considerable interest for conservation and management perspectives, especially in the current context of climate change and habitat fragmentation (Wasserman et al., 2012).

Studying the genetic structure is particularly suitable in (re)introduced populations as (re)introductions and translocations can have long‐term impacts on the genetic makeup of populations. They generally involve only a limited number of individuals thus retaining only a subset of the total genetic diversity of the source population, the occurrence of a strong genetic bottleneck being a likely event (Biebach & Keller, 2009; Hedrick, Gutierrez, & Lee, 2001). Nevertheless, a different origin of the founder individuals may genetically impact descendants for many generations (e.g., Biebach & Keller, 2009; Latch & Rhodes, 2005). Many studies have focused on the genetic consequences of introduction history (e.g., Barbanera et al., 2015; Biebach & Keller, 2009; Stephen et al., 2005), but the genetic consequences of (re)introductions and translocations remain difficult to study at the intrapopulation level due to several existing confounding factors (gene flow between translocated and other populations, unknown translocation history and founder genotypes, Mock, Latch, & Rhodes, 2004). However, as these processes represent the main tools within current conservation and management strategies (see Armstrong & Seddon, 2008; Batson et al., 2015; Latch & Rhodes, 2005), there is an essential need to assess the long‐term impacts of these strategies on the genetic structure of populations.

The fine‐scale spatial genetic structure of gregarious species is also strongly influenced by their social structures (Coltman, Pilkington, & Pemberton, 2003; Hazlitt et al., 2004; Storz, 1999). When individuals reproduce within social groups, the genetic differentiation among groups increases due to genetic drift (Storz, 1999). Concurrently, relatedness and inbreeding increase within groups and a socio‐spatial genetic substructuring of the population consequently appear. In mammals in which males are more prone to disperse than philopatric females (Greenwood, 1980), female behavior is thus expected to determine the spatial genetic structure of the population. There is often a stronger genetic structure in females than in males because of increased relatedness among spatially close individuals (e.g., for wild boars *Sus scrofa*, Podgórski, Scandura, & Jedrzejewska, 2014). Although socially mediated fine‐scale spatial genetic structure has been well characterized in various mammalian societies that exhibit stable social bonds (e.g., Hazlitt et al., 2004 in brush‐tailed rock‐wallabies *Petrogale penicillata*, Städele et al., 2015 in hamadryas baboons *Papio hamadryas*), studies are still scarce on species where group structure can be quite loose and characterized by fission–fusion dynamics, such as the large herbivores (but see Coltman et al., 2003 for Soay sheep *Ovis aries*, Archie et al., 2008 for African elephants *Loxodonta africana*).

To date, only a few studies have investigated the influence of the location of historic release sites on the current spatial genetic structure at the intrapopulation level (but see Simpson et al., 2013) and, to our knowledge, almost none concurrently assessed the relative contribution of the social‐spatial structure and the translocation history of the population. We tried to achieve this challenging task by studying an isolated Mediterranean mouflon (*Ovis gmelini musimon* × *Ovis* sp.) population in the Caroux‐Espinouse massif (southern France) 60 years after 19 founder individuals of diverse origins were introduced. Ungulates are especially well‐suited for such a task as most of their populations were (re)introduced worldwide during the last century (e.g., *Ovis canadensis mexicana*, Hedrick et al., 2001; Alpine ibex *Capra ibex ibex*, Biebach & Keller, 2009). Furthermore, these include social species with philopatric females composing matrilineal groups (e.g., wild boars *Sus scrofa*, Poteaux et al., 2009; Podgórski et al., 2014). Based on genetic data from 16 microsatellite loci combined with intensive visual and GPS monitoring of marked individuals, we thus aimed at disentangling the relative impacts of past introduction and socio‐spatial structure on the fine‐scale genetic structure of the population.

In the studied population, females are philopatric and establish their home range on their birth range (Dubois, Gerard, & Maublanc, 1992; Dubois et al., 1994; Dupuis et al., 2002). Regarding males, several forms of philopatry were previously observed. In relation to natal dispersal, dispersers and sedentary males (faithful to their birth range) have been observed (see Dubois, Quenette, & Bideau, 1993; Dubois et al., 1996; King & Brooks, 2003). Concerning reproductive dispersal, philopatric and unfaithful males have also been observed (Dubois et al., 1993, 1996). Additionally, individual movements and home ranges appeared to be strongly influenced by various natural and anthropogenic linear landscape features acting as behavioral barriers (Marchand et al., 2017). We expected these behavioral characteristics to lead to a strong socio‐spatial structure (see Garel et al., 2007; Martins et al., 2002), determining in turn the genetic structure of the population. In addition, we expected both structures to be sex‐specific due to the different use of space between males and females (Marchand et al., 2015, 2017) and the reproductive dispersal by males during the rutting period. Finally, the combination of marked socio‐spatial structure and geographic isolation of the population may magnify the historical genetic signature of introductions and be favorable to the maintenance of a genetic footprint (e.g., in red squirrels *Sciurus vulgaris*, Simpson et al., 2013).

## 2 MATERIALS AND METHODS

### 2.1 Study population, species, and data collection

**Figure 1:**
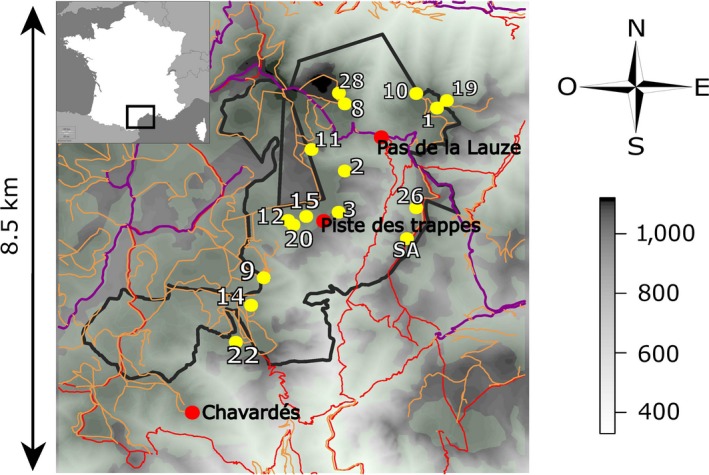
Map of the traps and sites of release in the Caroux‐Espinouse massif, southern France. The black line indicates the boundaries of the national fauna reserve, yellow points represent the traps of capture, red points represent the sites of release of founder individuals (see also Table 1), red lines represent hiking trails, orange lines represent tracks, and purple lines are the main roads crossing the study area. The gray scale indicates elevation in the massif (in meters), and green (uncolored) zones are closed/forested (open) areas

Data were collected in a National Hunting and Wildlife Reserve (1,658 ha, 532–1,124 m above sea level) in the Caroux‐Espinouse massif (43°38′N, 2°58′E, 17,000 ha, 130–1,124 m a.s.l., southern France; Figure 1). This low mountain area is characterized by deep valleys indenting plateaux and creating a mosaic of ridges and thalwegs (Marchand et al., 2017). Vegetation within the wildlife reserve is an irregular mosaic of beech, chestnut, and coniferous forests with open areas dominated by rocky slopes and broom and heather moorlands (Marchand et al., 2015). Human activities are strictly regulated in the wildlife reserve: Hunting is forbidden, and recreational activities are restricted to hiking on a few main trails (Marchand et al., 2014a).

Ewes of Mediterranean mouflon are commonly viewed as monotocous (twinning rate <3%) and sexually mature from 1.5 years of age (Garel et al., 2005). Births occur from late March to early June (Bon, Dardaillon, & Estevez, 1993). Although probably sexually mature at 2 years (see Festa‐Bianchet, 2012 in bighorn sheep), only rams three or more years old have been observed in rutting activities (Bon et al., 1992, 1995). Mating system is expected to be polygynous with a few large males achieving most of the mating (see Geist, 1971 for Dall's sheep *Ovis dalli*; see Jarman, 1983; Hogg, 1987 for Bighorn sheep *Ovis canadensis*). Females seem to be philopatric (Dubois et al., 1992, 1994; Dupuis et al., 2002; Martins et al., 2002; Maublanc et al., 1994) while males might tend to disperse as they get older (King & Brooks, 2003), although sedentary males have also been observed in this population (see Dubois et al., 1996). Mature males and females segregate most of the year but less during the rut as males join females to reproduce (Bon & Campan, 1989; Bon et al., 1992; Cransac et al., 1998; Dubois et al., 1993; Le Pendu et al., 1996). Faithfulness of males to their rutting site was also observed in this population (Dupuis et al., 2002; Martins et al., 2002).

The population of mouflon in the Caroux‐Espinouse massif originally grew from 19 Mediterranean mouflon released in the wildlife reserve between 1956 and 1960 (Table 1; Figure 1; Cugnasse, 1990; Garel et al., 2005). Since 1973, there has been hunting in surrounding unprotected areas from the beginning of September to the end of February (Garel et al., 2007) with approximately 500 mouflons of both sexes being harvested each year during the last decade. The population has been monitored by capture‐mark‐recapture since 1974. Mouflons were captured each year during spring using individual or collective traps and dropping nets baited with salt (Figure 1). Animals were marked with a numbered/colored collar; biometric measurements and hair samples were taken. Marked animals were visually monitored year‐round and spatially located on a map virtually divided into 125 × 125 m squares. Since 2006, some individuals have also been equipped with GPS collars (Lotek 3300S, revision 2; Lotek Engineering Inc., Carp, ON, Canada). GPS collars recorded animal locations using two different schedules: one recording locations intensively every 20 min during a restricted number of 48‐hr periods and one recording locations continuously every 2 hr (see Marchand et al., 2014b, 2015 for more details). All captures, handling, sampling, and collaring were performed according to the appropriate national laws for animal welfare, and procedures were approved by the pertinent administration. The nearest mouflon population is situated at 50 km away from the Caroux‐Espinouse massif, and a highway is separating the two areas. Accordingly with dispersal abilities of Mediterranean mouflon (see e.g., Dubois et al., 1996) and the impacts of anthropogenic linear landscape features on individual movements (Marchand et al., 2017), the Caroux‐Espinouse population is isolated and no natural gene flow could arise from other mouflon populations.

**Table 1:** Origin, year, site (see localizations on Figure 1), sex, and number of founder individuals released in the National Fauna Reserve of Caroux‐Espinouse massif (from Cugnasse & Houssin 1993 as cited in Garel, 2006)

Year	Origin	Site of release	Released individuals	
1956	France (Cadarache National Reserve)	Pas de la Lauze	2 ♀	2 ♂
1959	France (Cadarache National Reserve)	Chavardés	2 ♀	2 ♂
1960	The former Czechoslovakia	Pas de la Lauze	3 ♀	2 ♂
1960	France (Chambord National Domain)	Piste des trappes	3 ♀	3 ♂

### 2.2 Microsatellite genotyping

We used 262 hair samples from individuals trapped within a time period (2010–2014), that is short in comparison with the mouflon generation period (4.21 years, Hamel et al., 2016), preventing us from the existence of any temporal genetic structure. Analyzes were performed on individuals of both sexes having a fixed home range (in theory no longer expected to disperse), that is females two or more years old and males four or more years old (e.g., Dubois et al., 1992, 1993; Dupuis et al., 2002).

Genotyping was performed by the Antagene laboratory (Limonest, France, www.antagene.com). DNA extraction from hairs was performed on plates of 96 extraction columns (Nucleospin 96 Tissue, Macherey‐Nagel) in the presence of negative and positive extraction controls. The samples were lysed overnight at 56°C (according to manufacturer) and then DNA was purified and isolated using purification columns and vacuum filtration. DNA was then eluted to obtain one tube of 140 μl concentrated at 20–100 ng/μl and stored in numbered 96‐tubes plates in a freezer at −20°C. For each DNA sample, 16 microsatellite markers (see Supplementary A, Table S1) and two markers for sex identification (Amelogenin and ZFXY) were amplified by three multiplex polymerase chain reactions (PCR) and analyzed with an automated sequencer in two migrations. Each PCR was performed in a 8 μl final volume containing 4 μl of mastermix Taq Polymerase (Type‐It, Qiagen), respectively, 0.76, 0.38, and 0.58 μl of a pool of 8, 5, and 6 pairs of primers (for each of the three multiplex) at a concentration of 0.06–0.8 μm and 20 ng of genomic DNA to be amplified. One primer of each pair carried a fluorescent dye label. PCR amplifications were conducted in 96‐well microplates in dedicated post‐PCR area with negative air pressure allowing permanent air recycling using a T Gradient thermal cycler (Biometra) and consisted of an initial denaturing step (95°C, 5 min) followed by 35 cycles of denaturation (95°C, 30 s), annealing (58°C, 1 min 30 s) and extension (72°C, 30 s); cycles were followed by a final extension (60°C, 30 min). PCR products were resolved on a capillary sequencer ABI PRISM 3130 XL (Applied Biosystems) with formamide (denaturing conditions) and an internal size marker (600 Liz, Applied Biosystems). Migration conditions and genotypes of control samples were systematically checked. The electropherograms were analyzed using GENEMAPPER software (Applied Biosytems/Life Technologies) and analyzed independently by two analysts to determine the allele sizes for each marker of each individual. Reading errors were resolved, and ambiguous results were considered as missing data. Using such reading process, genotyping error rates were estimated to be <2% in our dataset (Queney et al., unpublished data). Individuals were genotyped between one and four times to obtain at least 13 markers with no missing data.

### 2.3 Genotyping errors

MICROCHECKER v.2.2.3 (Van Oosterhout et al., 2004) was used to detect null alleles. A Correspondence analysis (CA) was performed to identify and exclude outlier individuals. Data were checked for the presence of twin genotypes using the matching option in GenAlex v.6.501 (Peakall & Smouse, 2006, 2012). When detected, one of the two twin genotypes was randomly deleted to prevent bias in subsequent analyzes.

### 2.4 Genetic structure of the population

We performed sex‐specific analyzes (genetic and spatial), as different genetic and socio‐spatial structures between the sexes were expected due to (i) their different utilization of space (Marchand et al., 2015, 2017), (ii) the segregation of males and females most of the year (Cransac et al., 1998), and (iii) the philopatric behavior of females and much more mobile behavior of males (Dubois et al., 1992, 1993, 1996; Dupuis et al., 2002).

We first used a DAPC (Discriminant Analysis of Principal Component, library adegenet of R software, Jombart, Devillard, & Balloux, 2010; Jombart 2008a) to identify the most probable number of genetic clusters (*K*) in our population and assign each individual to a given cluster. *K* was determined by sequentially running a *K*‐means algorithm with increasing values of *K*. We used *K* ranging from 1 to 20 clusters and 1,000,000,000 iterations with 1,000 different starting values for each run of the algorithm. The clustering solutions were compared using the Bayesian Information Criterion (BIC). The optimal number of clusters was the one minimizing the BIC or, for competing solutions, the first one for which there was a marked break with the preceding BIC values. Once the optimal number of clusters had been identified, we used a cross‐validation procedure (iterated 10,000 times) to keep the optimal number of principal components (PCs) in the DAPC. The DAPC itself was then applied, performing first a Principal Component Analysis (PCA) on individual genotypes and then a Discriminant Analysis (DA) on principal components to assign each individual to a cluster with membership probabilities. We retained the minimal number of discriminant functions in order to maximize the total genetic variance explained. Genetic clustering reliability was assessed by looking at re‐assignment successes and proportions of individuals having membership probabilities lower than 0.80 for each genetic cluster.

In complement to the DAPC, we used a sPCA (Spatial Principal Component Analysis, library *adegenet*, Jombart et al., 2008b), to compare the spatially explicit patterns of genetic variability in our population with the socio‐spatial structure and spatial locations of founder events. The sPCA takes into account the spatial autocorrelation of allelic frequencies between neighbors (measured by Moran's index, *I*, Moran, 1948, 1950) and the genetic variability to describe the spatial genetic structure of the population. It maximizes the product of individuals' genetic variance and Moran's *I*. Permutation tests (*n* = 9,999) proposed by Jombart et al. 2008b were performed to assess the significance of the local and global spatial structures. The connection network was set using the inverse of the Euclidean pairwise distances between individuals. Animals were spatially assigned to the geographical coordinates of the arithmetic center of the trap/net where they were trapped. To compute trap arithmetic centers, we combined the visual resightings (*n* = 5,291) of 378 marked animals (304 adult females and 74 adult males) collected from 1990 to 2015 and GPS locations (*n* = 448,615) of 121 additional animals (81 females and 40 males) fitted with GPS collars from 2006 to 2016 and all having a fixed home range. Data from animals fitted with GPS were randomly subsampled to distribute evenly the number of GPS locations and visual resightings in the dataset. This step was performed to avoid an oversampling of a very local zone over the study area which may artificially bias the cluster analysis. We retrieved an average number of 20 locations for GPS females and males, both being in the order of magnitude of the average number of visual resightings per individual (females: 15.7 ± 16.6 (*SD*), males: 9.7 ± 8.9). Visual resightings had the advantage of providing rough spatial behavior from a large number of animals. Conversely, GPS data provided an intense and accurate monitoring (±24.5 m; Marchand et al., 2015) representative of habitats and time periods with very low (or null) visual detection probability (e.g., forested areas, nighttime) but concerned a more restricted number of individuals. We pooled both datasets to compute for each trap the arithmetic center of locations of all animals caught in that trap. A random spatial noise of a few meters using a uniform distribution was introduced to avoid getting duplicate coordinates for animals trapped at the same trap.

### 2.5 Socio‐spatial structure of the population

We defined the socio‐spatial units (groups of individuals living together) present in the population (Garel et al., 2007; Martins et al., 2002) using spatial data and arithmetic centers of traps (see above) to create groups of traps capturing individuals with a shared home range. This procedure (see Garel et al., 2007 for a similar approach in this population and Supplementary B for a reliability assessment of such an approach) allowed us to assign all genotyped animals (including the 22.8% having no visual resightings or GPS locations) to a given socio‐spatial unit depending on where they were trapped. Only traps having at least 30 locations (both visual resightings and GPS locations) were considered in the analyzes (median number of locations by trap and 2.5% and 97.5% quantiles for females: 263 [38–1150]_95%_; for males: 75 [40–270]_95%_). We then performed a hierarchical cluster analysis (with unweighted pair group method of aggregation) based on the dissimilarities among arithmetic centers (Euclidean distances) to identify relevant trap clusters. The number of socio‐spatial units selected by hierarchical clustering was set to be equal to the number of genetic clusters so we could assess if the genetic structure matched the socio‐spatial structure. Once the socio‐spatial units were defined, each genotyped individual was assigned to one of them based on its last trap of capture. Finally, we computed the proportion of individuals from each socio‐spatial unit assigned to the same genetic cluster to assess the extent to which socio‐spatial units and genetic clusters match.

### 2.6 Basic population genetics

Allele frequencies, number of alleles per locus (*N*_a_), allelic richness (*A*_r_, calculated using the rarefaction method (El Mousadik & Petit, 1996) to avoid bias due to variable sample sizes among different socio‐spatial units, and expected heterozygosity (*He* sensu Nei's gene diversity, Nei, 1973) were determined within each socio‐spatial unit for both sexes using FSTAT v.2.9.3.2 software (Goudet, 1995, 2001). Observed heterozygosity was determined using R software, package *hierfstat* (Goudet & Jombart, 2015). Departures from Hardy‐Weinberg Equilibrium (HWE) for each locus were tested using 10,000 randomizations in FSTAT. Similarly, *F*_is_ values per locus and socio‐spatial units were calculated, and their significance levels as compared to zero were assessed by randomizations of alleles among individuals within socio‐spatial units. Linkage disequilibrium was examined for all possible pairs of loci with exact *G*‐tests in FSTAT. *p*‐values were adjusted for multiple comparisons using the Bonferroni procedure when necessary (Bonferroni, 1936).

We computed global and pairwise *F*_st_ values (theta estimator, Weir & Cockerham, 1984) using FSTAT to measure the genetic differentiation in the population and between all possible pairs of socio‐spatial units. Significance levels of global *F*_st_ were assessed by exact‐G tests assuming random mating within samples, with 10,000 permutations. Significance levels of pairwise *F*_st_ values were assessed using permutations, and *p*‐values were adjusted for multiple comparisons using the Bonferroni procedure (Bonferroni, 1936).

### 2.7 Measurement of history footprint on the current population

We tested for the occurrence of recent bottlenecks (less than 15 generations ago, founder events) using the approach implemented in Bottleneck v.1.2.02 software (Cornuet & Luikart, 1996; Piry, Luikart, & Cornuet,^1999^) (within each socio‐spatial unit when relevant). When a population has faced a bottleneck, *H*_e_ is expected to be larger than expected heterozygosity under migration‐drift equilibrium (*H*_eq_) and a heterozygosity excess is detectable (Piry et al., 1999). Following the recommendations of Piry et al. (1999) for microsatellite datasets of less than 20 loci, we used the one‐step stepwise mutation (SMM) and the two‐phase (TPM) models of evolution. The TPM was parameterized with a variance among multiple steps of 12, 95% single step mutations and 5% multistep mutations. We performed 1,000 iterations and used Wilcoxon's signed rank test to assess the occurrence of heterozygote excess. Assumptions of sampling in a random mating and isolated population were respected (see Results 2.2).

All analyzes involving R packages were conducted with R 3.2.1 (R core team, 2016).

## 3 RESULTS

### 3.1 Genetic data

Among the genotyped samples, 237 individuals with fixed home range and known socio‐spatial unit had at least 13 successfully genotyped markers. Two outliers and five pairs of twins were identified among these individuals. Data on the 230 remaining individuals (69 males and 161 females) were considered in subsequent analyzes and were assigned to a socio‐spatial unit (see below). Comparison of the observed genotypes with the distribution of randomized genotypes generated with the program MICROCHECKER revealed that there were no null alleles in the dataset.

### 3.2 Genetic clustering

**Figure 2:**
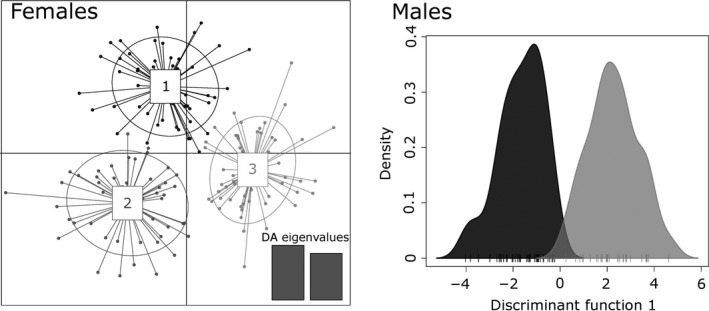
Genetic proximity among the 161 Mediterranean mouflon females and 69 males from the Caroux‐Espinouse area obtained by discriminant analysis of principal component (DAPC). For females (left), three genetic clusters and their 95% inertia ellipses are shown by different colors. Dots represent individuals while the number of discriminant analysis (DA) eigenvalues retained in the DAPC (number of axis) is shown at the bottom. For males (right), the two genetic clusters obtained are shown by different colors, and the tick marks are individuals

The DAPC revealed three or four genetic clusters for females (Figure 2; Supplementary material C, Figure S1). In the following, we kept *K* = 3 because the break in BIC values occurred between two and three clusters and *K* = 3 was the only clustering solution with nonoverlapping clusters (Figure 2). Cross‐validation procedures led us to keep the 10 first components of the PCA in subsequent DAPC. We then retained the two‐first discriminant functions of the DA which explained 50.7% of the total variance in the genetic data. Assignation successes within clusters were very high (98%, 96% and 100% for clusters 1, 2, and 3, respectively) and proportions of females assigned to a genetic cluster with membership probability inferior to 0.80 were relatively low (15%, 12%, and 10% for clusters 1, 2, and 3, respectively) meaning that the occurrence of three genetic clusters was well supported by the data.

For males, the optimal number of genetic clusters was *K* = 2 (Figure 2; Supplementary material C, Figure S2). We kept the first 10 PCs in the male DAPC and the first discriminant component of the DA which explained 58.3% of the total variance. Assignation success in each cluster reached 1 and membership probabilities were higher than 0.80 for all the males. Therefore, three genetic groups for females and two for males were identified in the population.

### 3.3 Spatial genetic structure

**Figure 3:**
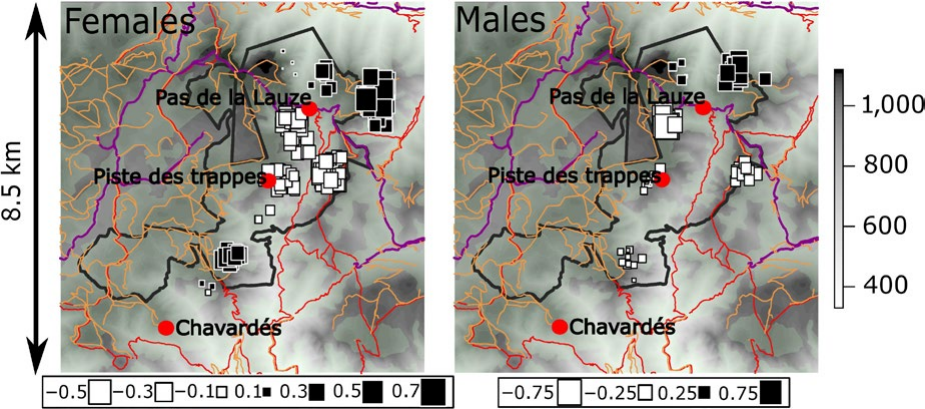
Geographic map of the first global lag scores of sPCA for females (left) and males (right) of the Mediterranean mouflon population of Caroux‐Espinouse massif. Individuals' scores are represented as squares with size proportional to the score, so that the maximum differentiation is between large black squares and large white squares. Red lines represent hiking trails, orange lines represent tracks, and purple lines are the main roads crossing the study area. The gray scale indicates elevation in the massif (in meters), and green (uncolored) zones are closed/forested (open) areas

The sPCA results corroborated those obtained with the DAPC. There was a significant global structure (global test, *p* = .003) for females, with only the first positive axis retained (Supplementary D) and no local structure (*p* = .84). The first sPCA axis accounted for most of the spatial genetic structure, covering a high proportion of the spatial autocorrelation and most of the variance in the genetic data (*I* = 0.27, var = 1.49). The first eigenvalue was 0.078 while the next eigenvalues were lower than 0.040 (Supplementary D). sPCA results supported marked genetic relatedness between females from the north and from the south, with females from the central part forming another genetic entity (Figure 3). Although sPCA visually opposed males from the north and from other parts of the wildlife reserve (Figure 3), this spatial opposition had no statistical support (global test: *p* = .48, local test: *p* = .26).

### 3.4 Socio‐spatial structure of the population

**Figure 4:**
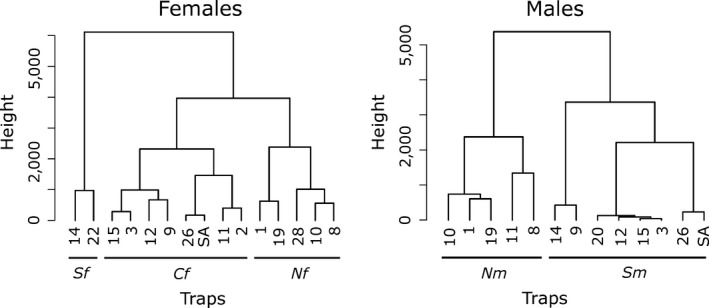
Cluster dendrograms resulting from hierarchical clustering applied on pairwise trap distances calculated based on relocation of individuals data (right: for females, left: for males). *Nf*,* Cf*,* Sf*,* Nm*, and *Sm*, correspond to socio‐spatial units as defined in the text

Based on the DAPC analyzes (see above), we set the number of socio‐spatial units in the hierarchical clustering of spatial data to three for females and to two for males. For females, the three socio‐spatial units corresponded to groups of traps located in the northern, central and southern parts of the study area (hereafter called “*Nf*,” “*Cf*,” and “*Sf*,” respectively; Figure 4). For males, one socio‐spatial unit grouped traps located in the northern (“*Nm*”) and the other in the southern part (“*Sm*”) of the study area (Figure 4). Traps in the central part of the reserve were principally grouped with traps from the north.

Socio‐spatial units of females and males in the population were at HWE as indicated by randomization tests (*p* = .99 and .19 for females and males, respectively) and confirmed by *F*_is_ values which were not significantly different from zero (Table 2 for overall loci values, Supplementary E, Tables S2 and S3 for per locus values). None of the pair of loci involved in the dataset were found to be in linkage disequilibrium (not shown). Despite a relatively low level of allelic richness, ranging from 3.42 ± 1.03 in socio‐spatial unit *Sf* to 3.74 ± 0.91 in socio‐spatial unit *Cf* (Table 2), the levels of heterozygosity were moderately high. The average *H*_e_ ranged from 0.56 ± 0.17 in the socio‐spatial unit *Sf* to 0.63 ± 0.12 in the socio‐spatial unit *Cf* and the average *H*_o_ ranged from 0.57 ± 0.21 in the socio‐spatial units *Sf* to 0.65 ± 0.13 in the socio‐spatial unit *Cf* (Table 2). The genetic differentiation among the different socio‐spatial units was low but significantly different from zero for females (global *F*_st_ = 0.023[0.011; 0.036]_95%_, *p* < .0001) while it was very low and not significantly different from zero for males (global *F*_st_ = −0.002[−0.007; 0.004]_95%_, *p* = .58). All pairwise *F*_st_ values were significantly different from zero only in females (Table 3).

**Table 2:** Sample size (*n*), number of allele (*N*_a_), allelic richness (*A*_r_), observed and expected heterozygosity (*H*_o_ and *H*_e_, respectively) averaged overall loci ± *SE*, and *F*_is_ values for the different socio‐spatial units of the Mediterranean mouflon population of Caroux‐Espinouse massif

Socio‐spatial unit	*n*	*N*_a_	*A*_r_	*H*_o_	*H*_e_	*F*_is_
Females						
*Nf*	37	3.75 ± 1.00	3.56 ± 0.99	0.61 ± 0.18	0.59 ± 0.17	−0.036
*Cf*	108	4.00 ± 0.89	3.74 ± 0.91	0.65 ± 0.13	0.63 ± 0.12	−0.038
*Sf*	16	3.44 ± 1.03	3.42 ± 1.03	0.57 ± 0.21	0.56 ±0.17	−0.003
Males						
*Nm*	36	3.80 ± 0.98	3.79 ± 0.98	0.59 ± 0.14	0.60 ± 0.14	0.029
*Sm*	33	3.88 ± 0.96	3.87 ± 0.96	0.61 ± 0.16	0.62 ± 0.14	0.007

None of the *F*_is_ values were significantly different from zero after Bonferroni correction (nominal levels: 0.00156 for males and 0.00104 for females).

**Table 3:** Pairwise *F*_st_ values between female and male socio‐spatial units of the Mediterranean mouflon population of the Caroux‐Espinouse massif

	Socio‐spatial unit	
	*Sf*	*Cf*
Females		
*Nf*	**0.0268**	**0.0164**
*Sf*		**0.0128**

	*Sm*	
Males		
*Nm*	−0.0017	

Values significantly different from zero are indicated in bold (adjusted nominal level after Bonferonni correction for females: 0.017, for males: 0.05).

Due to significant genetic differentiation between female socio‐spatial units, genetic bottleneck detection tests were performed within each socio‐spatial unit for females, but not for males. Except for the southern socio‐spatial unit of females (*Sf*), we found evidence supporting a recent bottleneck (<15 generations ago, founder events) for both sexes under both models of evolution (Table 4).

**Table 4:** Unilateral Wilcoxon tests *p*‐values of heterozygote excess detection tests (bottleneck detection test) performed for each sex in the Caroux‐Espinouse mouflon population (within each socio‐spatial unit as defined in the text for females)

	Socio‐spatial unit		
	*Nf*	*Cf*	*Sf*
Females			
T.P.M.	0.008	<0.001	*0.065*
S.M.M.	0.015	<0.001	*0.149*

	All socio‐spatial units
Males	
T.P.M.	<0.001
S.M.M.	<0.001

*n*, sample size; T.P.M., two‐phase model of evolution; S.M.M., stepwise mutation model. Nonsignificant values are indicated in italics.

### 3.5 Correspondence between socio‐spatial and genetic structures

Although no direct correspondence could be seen between socio‐spatial units and genetic clusters (Table 5), females from the north were genetically closer to females from the south of the wildlife reserve as evidenced by the sPCA (Figure 3; see above). Indeed, the largest proportion of females from socio‐spatial units *Nf* (56%) and *Sf* (64%) were grouped in genetic cluster 3 of the DAPC while females from socio‐spatial unit *Cf* were assigned to genetic cluster 1 (41%) or 2 (48%, Table 5). The admixture of the different socio‐spatial units observed in all genetic clusters indicated the occurrence of significant gene flow. For males, individuals from both socio‐spatial units were represented in equal proportions in both genetic clusters indicating a higher degree of admixture among males of the two socio‐spatial units than that observed in females.

**Table 5:** Proportion of individuals assigned with a membership probability higher than 0.80 from each socio‐spatial unit in each genetic cluster determined in DAPC

	Socio‐spatial unit		
	*Nf*	*Cf*	*Sf*
Females			
Genetic cluster 1	0.16	**0.41**	0.21
Genetic cluster 2	0.28	**0.48**	0.14
Genetic cluster 3	**0.56**	0.11	**0.64**

	*Nm*	*Sm*
Males		
Genetic cluster 1	**0.58**	0.48
Genetic cluster 2	0.42	**0.52**

Bold values represent the largest values for each socio‐spatial unit.

## 4 DISCUSSION

In males Mediterranean mouflon, the less philopatric sex, genetic structure was low compared to females for which the major axis of genetic structuring appeared to be a north‐south grouping (DAPC and sPCA). We also found a low but significant genetic differentiation between female socio‐spatial units (pairwise *F*_st_). However, this socio‐spatial organization effect was not detected by DAPC and sPCA suggesting that other processes, such as a residual effect of introduction history (e.g., Biebach & Keller, 2009), would be more in play than social factors in determining the genetic structure of females in this population.

### 4.1 Genetic diversity

As the isolated study population was introduced 60 years ago from 19 individuals, we could expect to observe low genetic diversity. Surprisingly, heterozygosity was not as low as we could expect due to the founder events and is comparable to the one reported in other Mediterranean mouflon populations (Corsica, Sardinia, or Central Italy, see Guerrini et al., 2015 and Supplementary material F, Tables S5, and S6). Similarly, Kaeuffer et al. (2007) revealed a higher than expected heterozygosity in another introduced mouflon population. Contrary to heterozygosity, allelic richness values in the study population were closer to those observed in the Cyprus population (see Supplementary Material F, Tables S5 and S6). Such low level of allelic richness could be a consequence of the founder events in our population. The results of bottleneck detection tests confirmed that genetic diversity is still influenced by past history in our population.

In introduced populations, the level of genetic diversity is supposed to increase with the number of founders, the degree of admixture in the founder group (mixed origin of founders results in the conservation of more diversity and a higher number of alleles) (Biebach & Keller, 2012; Maudet et al., 2002) and with *propagule pressure* (repeated introduction events prevent inbreeding) (Hufbauer et al., 2013). As the number and the genotypes of founder individuals determine the number of alleles (*N*_a_) present in the founded population, they have more detrimental effects on the number of alleles than on heterozygosity (Biebach & Keller, 2009, 2012; Kekkonen & Brommer, 2015). While *N*_a_ is mainly dependent on population size, heterozygosity is mainly impacted by the postbottleneck growth rate (Hedrick, 2011). In closed populations, *N*_a_ can only be restored by mutations; heterozygosity is thus expected to be restored faster and to be easier to save than *N*_a_ when conditions are favorable. In our population, founders from three different origins (Table 1) were released on four occasions, and growth rate after introduction is thought to have been as high as in other introduced mouflon populations (e.g., Kaeuffer et al., 2007) and most herbivore populations (Forsyth & Caley, 2006). These conditions could thus have favored heterozygosity maintenance at a relatively high level, comparable to unbottlenecked populations, while allelic richness still bears the scars of the founder events. Our results therefore support the theoretical work of Hufbauer et al. (2013) and Biebach and Keller (2012), and confirm that populations founded by multiple introductions of genetically diverse individuals avoid inbreeding effects.

### 4.2 Influence of socio‐spatial structure on genetic structure

We confirmed the previously highlighted low genetic differentiation between the different socio‐spatial units of the studied population (Petit et al., 1997). Global and all pairwise *F*_st_ values were nonetheless significantly different from zero for females, indicating genetic differentiation between socio‐spatial units, while a high degree of admixture between socio‐spatial units was observed for males. DAPC results showed a clear separation of genetic clusters which did not match with the socio‐spatial units, indicating that the socio‐spatial structure was not the main driver of the genetic structure in this population. Therefore, socio‐spatial structure contributed to the overall genetic structure of the population but other population processes contributed more (see below).

Differences in philopatric behavior between the sexes may lead to a more pronounced genetic structure in the philopatric sex (Goudet, Perrin, & Waser, 2002; Podgórski et al., 2014; Storz, 1999). As females showed a higher level of genetic structure than males (significant *F*_st_, more genetic clusters, and significant spatial genetic structure), our results confirm the philopatric behavior of females (Dubois et al., 1992, 1994; Martins et al., 2002) and suggest that gene flow is essentially male‐biased in this population, as evidenced also in Bighorn sheep (Boyce et al., 1999). Nevertheless, as in other mountain sheep species (Festa‐Bianchet, 1991), permanent natal dispersal of Mediterranean mouflon rams seems to be rare in our population (Dubois et al., 1996). Instead, rams have been shown to make excursions (temporary movement outside an established home range) during the rutting period (Marchand et al., unpublished data), and mating opportunities during these excursions may insure gene flow among the different socio‐spatial units. In the same vein, it was shown that while females show a stable movement pattern year round (moving preferentially toward familiar areas), males exhibit a more labile pattern and are more prone to move toward unfamiliar areas during the rutting period (Marchand et al., 2017; Martins et al., 2002). These sex‐specific behaviors lead to a higher spatial stability for females than for males. An interesting way to go further in the study of this sex‐biased reproductive dispersal would be to investigate mtDNA and Y‐chromosome, that is nonrecombining sex‐specific markers (Handley & Perrin, 2007).

### 4.3 Influence of introduction history on genetic structure

Surprisingly, given the spatial fidelity observed in female Mediterranean mouflon (Dubois et al., 1992, 1994; Martins et al., 2002) and a north‐south spatial disconnection (see Supplementary G), females from the north and the south were genetically closer to each other than to females from the central part of the reserve (see Table 5; Figure 3). Such a pattern would concur with the introduction history of the population: individuals introduced in the north of the reserve in 1956 and in the south of the reserve in 1959 all originated from the French Cadarache National Reserve (Table 1; Figure 1). This shared origin could therefore explain why individuals from the north and the south of the reserve are genetically close, while individuals from the central part of the reserve are thought to be an admixture of individuals from the former Czechoslovakia and the French Chambord National Domain. The higher genetic diversity (*H*_o_ and *H*_e_) in the *Cf* socio‐spatial unit (supposed mixed origin, see Table 2) and the still detectable founder effect strengthened this hypothesis. Although the release of individuals from the former Czechoslovakia in the north of the study area could explain why the historic genetic signal is relatively low, Czechoslovak individuals have most likely only dimly contributed to the genetic footprint as (i) they were released 4 years after Cadarache individuals, (ii) had a low survival probability (J. M. Cugnasse, pers. comm.), and (iii) the presence of behavioral barriers close to the release site (a road and a ridge) could have behaviorally isolated Czechoslovak individuals in the central part of the reserve.

Historical genetic signatures have been found to be persistent over numerous generations in other ungulate populations. For instance, in Creole cattle, Martínez et al. (2012) found a clear genetic signature of founder individuals more than 600 years after introduction. These results are in agreement with what was observed in Alpine ibex (*Capra ibex*), for which Biebach and Keller (2009) could genetically retrieve the introduction history of numerous populations founded during the 20th century. Similarly, DeYoung et al. (2003) find genetic similarities between source and transplanted white‐tailed deer (*Odocoileus virginianus*) populations regardless of their geographic separation. Finally, at intrapopulation and small geographic scale like in the present study, Simpson et al. (2013) showed that the site of release of founder individuals introduced at the end of the 19th century still has an impact on the current spatial genetic structure of the population of red squirrels. These results highlight that even after many generations, the genetic signature of source populations can still be present at intrapopulation scale. Moreover, female philopatry can be a factor explaining the persistence of this genetic signature in our population because global genetic structure (males and females considered in same analyzes) corresponds to female structure (results not shown).

## 5 CONCLUSION

Our findings highlighted important elements for wildlife management. First, despite the existence of male‐mediated gene flow, we showed that introduction history had long‐term consequences on intrapopulation spatial genetic structure, probably due to the high female philopatry which has favored the persistence of the historical genetic signature of introductions. These results confirm the importance for wildlife managers to account for sex‐specific spatial behaviors and gene flow when determining introduction/reinforcement strategies to ensure genetic mixing among introduced individuals from different sources. Indeed, as introductions often involve only a limited number of founders (Kekkonen & Brommer, 2015), genetic diversity may be low and a lack of genetic mixing between released individuals may result in introduction failure due to inbreeding depression and declining immunocompetence (see Armstrong & Seddon, 2008). Second, albeit allelic richness was relatively low, this study did not provide evidence that the Caroux‐Espinouse Mediterranean mouflon population shows depauperate genetic diversity. Such a result stresses the importance of choosing the right number, level of admixture (e.g., individuals from different origins) and place of release of individuals translocated when planning a (re)introduction program, to ensure that introduced genetic diversity expands to the entire population. Finally, the present study provided evidence that different levels of genetic structuring can co‐occur at fine spatial scale (1,700 ha) as compared to a mouflon home range (201 and 301 ha for females and males, respectively, Marchand, 2013). Such results would not have been obtained if sampling had been performed at a higher spatial resolution, highlighting the importance of designing sampling schemes in accordance with species biology and ecology. Additionally, such small‐scale spatial genetic structure suggests that small‐scale spatial population dynamic processes can occur in this population (see Coulson et al., 1999). All the results can be helpful for other populations, including endangered Corsican populations (*Ovis gmelini musimon* var. *Corsicana*) as they reveal important factors shaping the genetic structure of mouflon populations.

## CONFLICT OF INTEREST

All the authors disclose any potential sources of conflict of interest.

## DATA ACCESSIBILITY

Microsatellite and spatial data sets will be available from the Dryad Digital Repository.

## AUTHOR CONTRIBUTIONS

M.G., G.B., S.D., and D.M. conceptualized and designed the research. M.G., G.B., and P.M. conducted field sampling. J.A. conducted laboratory genetic analyzes. E.P., M.G., S.D., and P.M. conducted data analyzes. E.P., M.G., S.D., G.B., and P.M. interpreted the results. E.P., M.G., S.D., and G.B. wrote the manuscript with the help of all co‐authors.
