# Supplementary material for: Introduction history overrides social factors in explaining genetic structure of females in Mediterranean mouflon
Source: Ecol Evol. 2017 Nov 16;7(22):9580–91. doi: 10.1002/ece3.3433 (PMC5696436; doi:10.1002/ece3.3433)
Supplement: Supplementary file 1 [file ECE3-7-9580-s001.docx]

***Supplementary A:*** *Microsatellite description and Fluorescent DYE used for genotyping*

**Table S1: Microsatellite markers multiplex, size, fluorescent dyes and final concentrations used for genotyping of Mediterranean mouflon individuals from the Caroux-Espinouse massif population. The 3 different multiplex created are identified as “B”, “C” and “E”.**

| **Microsatellite locus** | **Reference** | **Genebank access number** | **Multiplex** | **Size (bp)** | **Fluorescent**  **Dye** | **Final concentration (µM)** |
| --- | --- | --- | --- | --- | --- | --- |
| **BM8125** | Bishop *et al.* (1994) | G18475 | B | 107-137 | Fam | 0.60 |
| **HUJ616** | Shalom *et al*. (1993) | M88250 | B | 121-167 | Ned | 0.20 |
| **OarCP34** | Ede *et al.* (1995) | U15699 | B | 115-137 | Vic | 0.06 |
| **OarFCB304** | Buchanan and Crawford (1993) | L01535 | B | 177-219 | Jaune | 0.20 |
| **OarJMP58** | Penty *et al.* (unpublished data) in Crawford *et al.* (1995) | U35058 | B | 124-176 | Pet | 0.20 |
| **OarVH72** | Pierson *et al.* (1993) | L12548 | B | 146-174 | Fam | 0.20 |
| **SRCRSP1** | Arevalo *et al.* (1994) | L22192 | B | 147-181 | Vic | 0.10 |
| **INRA063** | Vaiman *et al.* (1994) | X71507 | C | 161-185 | Ned | 0.40 |
| **MAF65** | Buchanan *et al*. (1992) | M67437 | C | 121-151 | Ned | 0.20 |
| **MCM140** | Hulme *et al.* (1995) | L38979 | C | 194-220 | Ned | 0.40 |
| **OarFCB193** | Buchanan and Crawford (1993) | L01533 | C | 93-143 | Bleu | 0.08 |
| **OarJMP29** | Penty *et al.* (unpublished data) in Crawford *et al.* (1995) | U30893 | C | 103-157 | Vic | 0.10 |
| **MAF70** | Buchanan and Crawford (1992) | M77199 | E | 153-195 | Pet | 0.20 |
| **OarFCB226** | Buchanan *et al.* (1994) | L20006 | E | 153-187 | Fam | 0.40 |
| **OarHH47** | Henry *et al.* (1993) | L12557 | E | 167-199 | Vic | 0.20 |
| **SRCRSP9** | Bhebhe *et al*. (1994) | L22201 | E | 105-143 | Pet | 0.40 |
| **Amelogenin** | Developed by Antagène laboratory | NA | E | 214 & 259 | Pet | 0.40 |
| **ZFXY** | Developed by Antagène laboratory | NA | E | 275 & 279 | Fam | 0.20 |

***Supplementary B:*** *Is the trap-based socio-spatial unit a good approximation of the individual-based one?*

Since spatial data were not available for all genotyped individuals, we used a trap-based approach (see Garel *et al.* 2007 for a similar approach) to define socio-spatial units present in the population (trap-based socio-spatial units). As explained in the main article, this approach allowed us to assign all genotyped animals (including the 22.8% having no visual resightings or GPS locations) to a given socio-spatial unit depending on where they were trapped.

To assess the reliability of this approach, we wondered if all animals captured at a certain trap belong to the same socio-spatial unit. To answer this question, we computed groups of individuals living together (individual-based socio-spatial units) using hierarchical clustering analysis (UPGMA, see main article for details) on individuals resighted at least four times or GPS collared (n=394, same results being obtained using a threshold of 10 spatial locations, see Martins *et al.* 2002). The objective was to assess if the socio-spatial units obtained were the same whether analyses were performed at the individual scale or at the trap scale, aggregating all spatial locations of individuals captured at the same trap.

Results (Table S2) showed that correspondence was greater than 94.7% for females and 70% for males, suggesting that it is possible, especially for females, to assign individuals to a socio-spatial unit based on their trap of capture. The lower correspondence between individual- and trap-based socio-spatial units observed for males indicates that males are more mobiles than females.

**Table S2: Number of individuals from each individual-based cluster captured in each trap-based cluster. UPGMA was based on Euclidean distances between averaged locations of each animal (individuals-based analysis) or of each trap (trap-based analysis). Red values represent the maximal value of individuals from the same individual-based cluster belonging to the same trap-based cluster.**

|  |  | **Trap-based cluster** | | |
| --- | --- | --- | --- | --- |
|  |  | ***Nf*** | ***Cf*** | ***Sf*** |
| **Females individual-based cluster** | **1** | 97 | 6 | 0 |
|  | **2** | 1 | 184 | 1 |
|  | **3** | 0 | 0 | 18 |
|  |  | ***Nm*** | ***Sm*** |  |
| **Males individual-based cluster** | **1** | 47 | 28 |  |
|  | **2** | 0 | 12 |  |

***Supplementary C:*** *Defining genetic clusters thanks to a DAPC.*

**Figure S1: BIC values associated with each K value tested from the K-means procedure of the DAPC for females in the Caroux-Espinouse mouflon population. It indicated as an optimal number of genetic cluster of K = 3 or K = 4.**

**Figure S2: BIC values associated with each K value tested from the K-means procedure of the DAPC for males in the Caroux-Espinouse mouflon population. It indicates an optimal number of cluster of K = 2.**

***Supplementary D:*** *sPCA axis selection.*

**Figure S3: sPCA eigenvalues for females of the Mediterranean mouflon population of the Caroux-Espinouse massif.**

***Supplementary E:*** *Genetic variability per locus.*

**Table S3: Genetic variability per locus as computed for each socio-spatial unit of females and *Fis* values. Legend: n: sample size; *n_a_*: number of alleles; *Ar*: allelic richness; *Ho*: observed heterozygosity; *He*: expected heterozygosity.**

|  | ***Nf*** | | | | | | ***Cf*** | | | | | | ***Sf*** | | | | | |
| --- | --- | --- | --- | --- | --- | --- | --- | --- | --- | --- | --- | --- | --- | --- | --- | --- | --- | --- |
| **Locus** | ***n*** | ***n_a_*** | ***A_r_*** | ***Ho*** | ***He*** | ***Fis*** | ***n*** | ***n_a_*** | ***A_r_*** | ***Ho*** | ***He*** | ***Fis*** | ***n*** | ***n_a_*** | ***A_r_*** | ***Ho*** | ***He*** | ***Fis*** |
| **BM8125** | 36 | 5 | 4.98 | 0.750 | 0.754 | 0.006 | 105 | 5 | 4.98 | 0.848 | 0.781 | -0.085 | 16 | 5 | 5.00 | 0.750 | 0.719 | -0.043 |
| **HUJ616** | 37 | 3 | 3.00 | 0.703 | 0.557 | -0.262 | 108 | 4 | 3.64 | 0.657 | 0.608 | -0.081 | 16 | 3 | 2.94 | 0.688 | 0.500 | -0.375 |
| **INRA063** | 35 | 2 | 2.00 | 0.486 | 0.481 | -0.010 | 104 | 2 | 2.00 | 0.539 | 0.502 | -0.073 | 16 | 2 | 2.00 | 0.750 | 0.508 | -0.475 |
| **MAF65** | 37 | 2 | 1.65 | 0.054 | 0.053 | -0.014 | 108 | 3 | 2.14 | 0.315 | 0.317 | 0.005 | 16 | 2 | 1.94 | 0.063 | 0.063 | 0.000 |
| **MAF70** | 37 | 5 | 4.95 | 0.622 | 0.712 | 0.127 | 107 | 5 | 4.80 | 0.710 | 0.663 | -0.071 | 15 | 4 | 4.00 | 0.533 | 0.552 | 0.034 |
| **MCM140** | 36 | 4 | 4.00 | 0.778 | 0.734 | -0.060 | 107 | 4 | 3.98 | 0.729 | 0.713 | -0.023 | 16 | 4 | 4.00 | 0.688 | 0.773 | 0.111 |
| **OarCP34** | 37 | 3 | 3.00 | 0.649 | 0.645 | -0.005 | 108 | 4 | 3.26 | 0.574 | 0.567 | -0.012 | 16 | 3 | 3.00 | 0.438 | 0.604 | 0.276 |
| **OarFCB193** | 37 | 5 | 4.38 | 0.784 | 0.706 | -0.109 | 108 | 5 | 4.66 | 0.741 | 0.713 | -0.040 | 16 | 5 | 4.88 | 0.438 | 0.679 | 0.356 |
| **OarFCB226** | 37 | 4 | 3.95 | 0.703 | 0.643 | -0.093 | 108 | 4 | 3.87 | 0.639 | 0.665 | 0.039 | 16 | 3 | 3.00 | 0.688 | 0.613 | -0.122 |
| **OarFCB304** | 37 | 4 | 3.68 | 0.676 | 0.588 | -0.149 | 106 | 4 | 3.90 | 0.557 | 0.579 | 0.038 | 16 | 3 | 2.94 | 0.313 | 0.467 | 0.330 |
| **OarHH47** | 37 | 4 | 3.65 | 0.676 | 0.611 | -0.106 | 108 | 4 | 3.86 | 0.630 | 0.617 | -0.021 | 16 | 4 | 4.00 | 0.625 | 0.598 | -0.045 |
| **OarJMP29** | 36 | 4 | 3.41 | 0.444 | 0.494 | 0.100 | 107 | 5 | 4.06 | 0.673 | 0.692 | 0.028 | 16 | 4 | 4.00 | 0.688 | 0.675 | -0.019 |
| **OarJMP58** | 37 | 5 | 4.88 | 0.730 | 0.708 | -0.031 | 108 | 5 | 4.96 | 0.833 | 0.764 | -0.090 | 16 | 5 | 4.998 | 0.750 | 0.760 | 0.014 |
| **OarVH72** | 37 | 3 | 2.65 | 0.488 | 0.515 | 0.056 | 106 | 3 | 2.95 | 0.642 | 0.557 | -0.151 | 16 | 2 | 2.000 | 0.500 | 0.508 | 0.016 |
| **SRCRSP1** | 37 | 3 | 2.93 | 0.568 | 0.553 | -0.027 | 108 | 3 | 2.87 | 0.593 | 0.557 | -0.064 | 16 | 3 | 2.998 | 0.813 | 0.565 | -0.439 |
| **SRCRSP9** | 36 | 4 | 3.805 | 0.6111 | 0.631 | 0.031 | 107 | 4 | 3.998 | 0.7383 | 0.737 | -0.001 | 15 | 3 | 3.000 | 0.3333 | 0.443 | 0.247 |

**Table S4: Genetic variability per locus as computed for each socio-spatial unit of males. Legend: n: sample size*; n_a_*: number of alleles; *Ar*: allelic richness; *Ho*: observed heterozygosity; *He*: expected heterozygosity.**

|  | ***Nm*** | | | | | | ***Sm*** | | | | | |
| --- | --- | --- | --- | --- | --- | --- | --- | --- | --- | --- | --- | --- |
| **Locus** | ***n*** | ***n_a_*** | ***A_r_*** | ***Ho*** | ***He*** | ***Fis*** | ***n*** | ***n_a_*** | ***A_r_*** | ***Ho*** | ***He*** | ***Fis*** |
| **BM8125** | 36 | 5 | 5.00 | 0.722 | 0.799 | 0.096 | 31 | 5 | 5.00 | 0.807 | 0.780 | -0.034 |
| **HUJ616** | 36 | 4 | 3.72 | 0.417 | 0.435 | 0.041 | 33 | 4 | 3.94 | 0.546 | 0.545 | -0.002 |
| **INRA063** | 35 | 2 | 2.00 | 0.543 | 0.500 | -0.086 | 33 | 2 | 2.00 | 0.394 | 0.509 | 0.227 |
| **MAF65** | 36 | 2 | 2.00 | 0.250 | 0.221 | -0.129 | 33 | 2 | 2.00 | 0.182 | 0.217 | 0.162 |
| **MAF70** | 36 | 5 | 5.00 | 0.640 | 0.669 | 0.045 | 32 | 5 | 5.00 | 0.719 | 0.694 | -0.036 |
| **MCM140** | 36 | 4 | 4.00 | 0.667 | 0.701 | 0.049 | 33 | 4 | 4.00 | 0.758 | 0.717 | -0.057 |
| **OarCP34** | 36 | 3 | 3.00 | 0.583 | 0.633 | 0.079 | 33 | 4 | 3.94 | 0.636 | 0.592 | -0.075 |
| **OarFCB193** | 36 | 5 | 5.00 | 0.861 | 0.702 | -0.226 | 33 | 5 | 5.00 | 0.758 | 0.699 | -0.083 |
| **OarFCB226** | 36 | 4 | 3.98 | 0.611 | 0.617 | 0.010 | 33 | 4 | 4.00 | 0.667 | 0.711 | 0.063 |
| **OarFCB304** | 35 | 4 | 4.00 | 0.657 | 0.643 | -0.022 | 33 | 4 | 4.00 | 0.546 | 0.656 | 0.168 |
| **OarHH47** | 36 | 4 | 4.00 | 0.667 | 0.668 | 0.002 | 33 | 4 | 4.00 | 0.727 | 0.674 | -0.079 |
| **OarJMP29** | 36 | 4 | 3.98 | 0.500 | 0.593 | 0.157 | 33 | 4 | 4.00 | 0.758 | 0.679 | -0.116 |
| **OarJMP58** | 36 | 5 | 5.00 | 0.556 | 0.727 | 0.236 | 33 | 5 | 5.00 | 0.636 | 0.771 | 0.174 |
| **OarVH72** | 35 | 3 | 3.00 | 0.514 | 0.472 | -0.090 | 32 | 3 | 2.97 | 0.469 | 0.469 | 0.000 |
| **SRCRSP1** | 36 | 3 | 3.00 | 0.500 | 0.542 | 0.078 | 33 | 3 | 3.00 | 0.546 | 0.552 | 0.012 |
| **SRCRSP9** | 33 | 4 | 4.00 | 0.697 | 0.741 | 0.060 | 32 | 4 | 4.00 | 0.688 | 0.634 | -0.085 |

***Supplementary F:*** *Allelic richness and observed heterozygosity calculated on the four loci in common with Guerrini et al. (2015).*

**Table S5: Averaged allelic richness (*Ar*) and observed heterozygosity (*Ho*) calculated on the four loci in common with Guerrini *et al.* (2015) (i.e. SRCRSP9, MAF70, OarJMP58 and OarFCB304) for Mediterranean mouflon from the Caroux-Espinouse massif population.**

|  | |  |  | | | ***Socio-spatial units*** | |  |
| --- | --- | --- | --- | --- | --- | --- | --- | --- |
| ***Females*** | |  | ***Nf*** | | | ***Cf*** | | ***Sf*** |
|  | | ***Ar*** | 4.33 | | | 4.41 | | 3.73 |
|  |  | ***Ho*** | 0.66 | | | 0.71 | | 0.48 |
| ***Males*** |  | | |  | ***Nm*** | | ***Sm*** | |
|  | ***Ar*** | | |  | 4.5 | | 4.5 | |
|  | ***Ho*** | | |  | 0.64 | | 0.65 | |

**Table S6: Allelic richness (*Ar*) and observed heterozygosity (*Ho)* from Guerrini *et al.* (2015) for the four loci in common with the present study.**

|  | ***Corsica*** | | ***Sardinia*** | | ***Central Italy*** | | ***Cyprus*** | |
| --- | --- | --- | --- | --- | --- | --- | --- | --- |
| ***Locus*** | ***Ar*** | ***Ho*** | ***Ar*** | ***Ho*** | ***Ar*** | ***Ho*** | ***Ar*** | ***Ho*** |
| **MAF70** | 7.8 | 0.53 | 5.8 | 0.60 | 4.9 | 0.67 | 2.3 | 0.08 |
| **OarFCB304** | 8.0 | 0.63 | 7.2 | 0.85 | 6.4 | 0.45 | 3.7 | 0.51 |
| **OarJMP58** | 10.2 | 0.58 | 6.9 | 0.75 | 8.4 | 0.59 | 2.4 | 0.35 |
| **SR-CRSP9** | 6.7 | 0.63 | 3.9 | 0.74 | 6.0 | 0.64 | 1.0 | Monomorphic |
| ***Mean*** | 8.18 | 0.59 | 5.95 | 0.735 | 6.43 | 0.59 | 2.35 | 0.24 |

***Supplementary G:*** *Spatial disconnection between individuals from the north and the south of the reserve.*

Females from the north and the south of the study area were genetically closer to each other than to females from the central part of the reserve (see main article). One possible explanation could be that gene flows are favored between the northern and the southern female socio-spatial units.

To answer this question we used spatial data to measure spatial disconnection between the different female socio-spatial units. We assessed if females from the north visit the southern part of the study area more frequently than the central part. Additionally, in order to see if males ensure gene flow preferentially between northern and southern female socio-spatial units (explaining the genetic proximity between these two socio-spatial units), we assigned male individuals to socio-spatial units as defined thanks to female data (trap clustering, see main article, Figure 4).

*Based on GPS data.*

In order to assess more precisely the spatial disconnection existing between the different female socio-spatial units, we performed analyses based on GPS data. 61 adult females and 27 males were monitored by GPS and associated with at least 300 GPS locations (12 females and 8 males in *Nf*, 33 females and 14 males in *Cf*, 12 females and 5 males is *Sf*). For each individual, 300 GPS locations were randomly sampled. GPS locations of all monitored individuals from each socio-spatial unit were pooled and used to calculate the utilization distribution for each socio-spatial unit using the Brownian Bridge Movement Model (BBMM, Horne *et al*. 2007). The smoothing parameter “sig1” (Brownian motion variance) was estimated for each socio-spatial unit using the likelihood approach developed by Horne *et al.* (2007), and “sig2”, the GPS location error was fixed at 24.5m (see Marchand *et al.* 2015). We then calculated overlap between all pairs of socio-spatial unit home ranges (volume of intersection between the two UDs). The spatial analyses were performed using the R package *adehabitatHR* (Calenge 2006).

Table S7: Overlap among the home ranges of different female socio-spatial units based on female or on male data.

|  | | ***Females*** | |
| --- | --- | --- | --- |
|  | ***Nf*** | | ***Cf*** |
| ***Cf*** | 0.198 | |  |
| ***Sf*** | 0.003 | | 0.150 |
|  | | ***Males*** | |
|  | ***Nf*** | | ***Cf*** |
| ***Cf*** | 0.191 | |  |
| ***Sf*** | 0.024 | | 0.176 |

We can see in Table S7 that for females, northern and southern socio-spatial units almost never overlap (0.3%) as compared to northern and central socio-spatial units (19.8%) or southern and central socio-spatial units (15%). For males, similar results were obtained, since only 2.4% of the home ranges of individuals assigned to the *Nf* socio-spatial unit overlapped those of males assigned to *Sf*. It indicated that no preferential movements occur between northern and southern female socio-spatial units (neither for females nor for males) and that northern and southern female socio-spatial units are spatially disconnected.

*Based on capture data.*

698 adult individuals (503 females and 195 males) were (re)captured between 1990 and 2015. Among these individuals, only 79 were sequentially captured at different traps (55 females and 24 males) while 169 were captured several times at the same trap. Among the females captured at different traps during their lifetimes only three were captured at a trap in a different socio-spatial unit (see trap clustering, see main article, Figure 4). Among these three females, two switched between the *Nf* and the *Cf* socio-spatial unit and one switched between the *Sf* and the *Cf* socio-spatial units. Regarding males, one individual switched between *Sf* and *Cf* while another switched between *Nf* and *Cf*. *Nf* and *Sf* female socio-spatial units were thus completely disconnected.

***References***

Arevalo, E. et al., 1994. Caprine microsatellite dinucleotide repeat polymorphisms at the SR-CRSP-1, SR-CRSP-2, SR-CRSP-3, SR-CRSP-4 and SR-CRSP-5 loci. *Animal genetics*, 25(3), p.202.

Bhebhe, E. et al., 1994. Caprine microsatellite dinucleotide repeat polymorphisms at the SR-CRSP-6, SR-CRSP-7, SR-CRSP-8, SR-CRSP-9 and SR-CRSP-10 loci. *Animal genetics*, 25, p.203.

Bishop, M.D. et al., 1994. A genetic linkage map for cattle. *Genetics*, 136(2), pp.619–639.

Buchanan, F.C. & Crawford, A.M., 1992. Ovine dinucleotide repeat polymorphism at the MAF70 locus. *Animal Genetics*, 23, p.185.

Buchanan, F. C., Swarbrick, P. A. & Crawford, A. M., 1992. Ovine dinucleotide repeat polymorphism at The W65 Locus. *Animal Genetics*. **23**, 85.

Buchanan, F.C. & Crawford, A.M., 1993. Ovine microsatellites at the OarFCB11, OarFCBl28,OarFCB193,OarFCB266 and OarFCB304 loci. *Animal Genetics*, 24(2), p.145.

Buchanan, F.C., Galloway, S.M. & Crawford, A.M., 1994. Ovine microsatellites at the OarFCB5 , OarFCB19,OarFCB20,OarFCB48,OarFCB129 and OarFCB226 loci. *Animal genetics*, 25, p.60.

Buchanan, F.C., Swarbrick, P.A. & Crawford, A.M., 1992. Ovine dinucleotide repeat polymorphism at the W65 locus. *Animal Genetics*, 23, p.85.

Calenge, C., 2006. The package “adehabitat” for the R software: A tool for the analysis of space and habitat use by animals. *Ecological Modelling*, 197(3–4), pp.516–519.

Crawford, A.M. et al., 1995. An Autosomal Genetic Linkage Map of the Sheep Genome. *Genetics*, 140, pp.703–724.

Ede, A.J., Pierson, C.A. & Crawford, A.M., 1995. Ovine microsatellites at the OarCP34, OarCP38, OarCP43, OarCP49,OarCP73,OarCP79 and OarCP99 loci. *Animal Genetics*, 26, pp.130–131.

Garel, M. et al., 2007. Selective harvesting and habitat loss produce long-term life history changes in a mouflon population. *Ecological Applications*, 17(6), pp.1607–1618.

Guerrini, M. et al., 2015. Molecular DNA identity of the mouflon of Cyprus (Ovis orientalis ophion , Bovidae): Near Eastern origin and divergence from Western Mediterranean conspecific populations. *Systematics and Biodiversity*, 13(5), pp.472–483.

Henry, H.M. et al., 1993. Ovine microsatellites at the OarHH35, OarHH41, OarHH44, OarHH47 and OarHH64 loci. *Animal Genetics*, 24(3), pp.222–222.

Horne, J.S. et al., 2007. Analyzing animal movements using Brownian bridges. *Ecology*, 88(9), pp.2354–2363.

Hulme, D.J. et al., 1995. Polymorphic sheep microsatellites at the McM2, McM131, McM135, McMl36, McM140, McM200, McM214, McM373, McM505, McM507 and McM522 loci. *Animal Gene*, 26, pp.369–370.

Marchand, P. et al., 2015. Coupling scale-specific habitat selection and activity reveals sex-specific food/cover trade-offs in a large herbivore. *Animal Behaviour*, 102, pp.169–187.

Pierson, C.A. et al., 1993. Ovine microsatellites at the OarVH34,OarVH58,OarVH61 and OarVH72 loci. *Animal Genetics*, 24, p.224.

Shalom, A., Soller, M. & Friedman, A., 1993. Dinucleotide repeat polymorphism at the bovine HUJ616 locus. *Animal Genetics*, 24, p.327.

Vaiman, D. et al., 1994. A set of 99 cattle microsatellites: characterization, synteny mapping, and polymorphism. *Mammalian Genome*, 5(5), pp.288–297.
